# Múltiplas Metástases Intracardíacas – Um Coração Rosário

**DOI:** 10.36660/abc.20230732

**Published:** 2024-07-01

**Authors:** Mariana Tinoco, Margarida Castro, Hans Dabó, Filipa Cordeiro, Pedro von Hafe, António Lourenço

**Affiliations:** 1 Unidade Local de Saúde do Alto Ave Guimarães Portugal Unidade Local de Saúde do Alto Ave, Guimarães – Portugal

**Keywords:** Cardio-Oncologia, Metastases Neoplasicas, Imagem Multimodal, Diagnóstico Por Imagem/Métodos, Ecocardiografia/métodos

**Figura 1:**
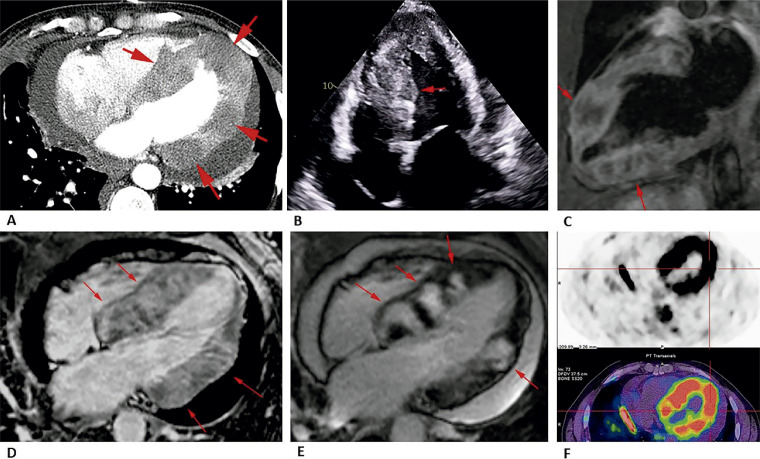
– Diferentes modalidades de imagem evidenciando múltiplas metástases intracardíacas. A) TC de tórax; B) Ecocardiografia transtorácica; CE) RM cardíaca mostrando lesões hipointensas nas imagens ponderadas em T2 com halo periférico de hiperintensidade (C); realce heterogêneo precoce (D) e realce tardio intenso com gadolínio (E); F) captação anormal de fluorodesoxiglicose no 18F-FDG PET/CT.

Um homem de 52 anos estava sendo submetido a quimioterapia de primeira linha com pemetrexedo devido a um adenocarcinoma de pulmão hilar direito em estágio IV com prováveis metástases pleurais, pericárdicas, hepáticas e ósseas. Durante o acompanhamento, nenhuma evidência de progressão da doença foi encontrada. Quatro anos depois, uma tomografia computadorizada de tórax de acompanhamento documentou um realce nodular heterogêneo do miocárdio do ventrículo esquerdo (VE) (Figura 1A), as lesões restantes estavam sobrepostas. Ele não apresentava sintomas cardiovasculares e o exame clínico era normal. A ecocardiografia transtorácica (Figura 1B) e a ressonância magnética cardíaca (Figuras 1C-E) revelaram diversas massas endomiocárdicas bem arredondadas, algumas das quais multilobuladas, por vezes com extensão quase transmural. As lesões distribuem-se predominantemente ao longo da parede inferoseptal do VE, parede ínferolateral, parede anterolateral, parede anterior e lado direito do septo interventricular. Essas lesões apresentavam características de sinal heterogêneas, sendo predominantemente hiperintensas nas imagens ponderadas em T1, hipointensas nas imagens ponderadas em T2 com halo periférico de hiperintensidade, mostrando realce precoce heterogêneo e tardio intenso por gadolínio; e captação anormal de fluorodesoxiglicose em PET/CT com 18F-FDG (Figura 1F).

Assim, embora as massas cardíacas não tenham sido biopsiadas, o seu aspecto imagiológico foi preocupante para a progressão tumoral. Decidiu-se adicionar pembrolizumab ao pemetrexedo. Atualmente, o paciente encontra-se vivo há mais de 18 meses após o diagnóstico de metástases cardíacas.

Múltiplas metástases cardíacas de câncer de pulmão de células não pequenas são extremamente raras e muitas vezes não são diagnosticadas até a morte. O prognóstico é uniformemente ruim, com poucos sobreviventes a longo prazo.^1^
