# Supplementary material for: Múltiplas Metástases Intracardíacas – Um Coração Rosário
Source: Arq Bras Cardiol. 2024 Jul 1;121(6):e20230732. [Article in Portuguese] doi: 10.36660/abc.20230732 (PMC12080609; doi:10.36660/abc.20230732)
Supplement: Translation

# Translation: Multiple Intracardiac Metastases – A Chaplet Heart

**DOI:** 10.36660/abc.20230732i

**Authors:** Mariana Tinoco, Margarida Castro, Hans Dabó, Filipa Cordeiro, Pedro von Hafe, António Lourenço

**Affiliations:** 1 Portugal Unidade Local de Saúde do Alto Ave, Guimarães – Portugal

**Keywords:** Cardioncology, Neoplasm Metastasis, Multimodal Imaging, Diagnostic Imaging/methods, Echocardiography/methods

A 52-year-old male was undergoing first-line chemotherapy with pemetrexed due to a stage IV right hilar lung adenocarcinoma with likely pleural, pericardial, liver, and bone metastases. During follow-up, no evidence of disease progression was found. Four years later a follow-up chest CT scan documented a nodular heterogeneous enhancement of the left ventricular (LV) myocardium (Figure 1A), the remaining lesions were overlapping. He had no cardiovascular symptoms and clinical examination was unremarkable. Transthoracic echocardiography (Figure 1B) and cardiac MRI (Figures 1C-E) revealed several well-rounded endo-myocardial masses, some of which multilobulated sometimes with almost transmural extension. The lesions are predominantly distributed along the LV inferoseptal wall, inferolateral wall, anterolateral wall, anterior wall, and right side of the interventricular septum. These lesions had heterogeneous signal characteristics, being predominantly hyperintense on T1-weighted images, hypointense on T2-weighted images with a peripheral halo of hyperintensity, showing early heterogeneous and late intense gadolinium enhancement; and abnormal uptake of fluorodeoxyglucose in a 18F-FDG PET/CT (Figure 1F).

**Figure 1:** – Different imaging modalities showing multiple intracardiac metastases. A) Chest CT; B) Transthoracic echocardiography; C-E) Cardiac MRI showing hypointense lesions on T2-weighted images with a peripheral halo of hyperintensity (C); early heterogeneous enhancement (D) and late intense gadolinium enhancement (E); F) abnormal uptake of fluorodeoxyglucose on 18F-FDG PET/CT.

Thus, though the cardiac masses were not biopsied, their imagiological appearance was concerning for tumor progression. It was decided to add pembrolizumab to pemetrexed. Currently, the patient is alive over 18 months after the diagnosis of cardiac metastases.

Multiple cardiac metastases from non-small cell lung cancer are extremely rare and often go undiagnosed until postmortem. The prognosis is uniformly poor, with few long-term survivors.^1^
